# Easy or Not—The Advances of EZH2 in Regulating T Cell Development, Differentiation, and Activation in Antitumor Immunity

**DOI:** 10.3389/fimmu.2021.741302

**Published:** 2021-10-19

**Authors:** Jiaqi Huang, Jie Zhang, Zhengyang Guo, Chen Li, Zhen Tan, Junjie Wang, Jianling Yang, Lixiang Xue

**Affiliations:** ^1^ Department of Radiation Oncology, Peking University Third Hospital, Beijing, China; ^2^ Center of Basic Medical Research, Institute of Medical Innovation and Research, Peking University Third Hospital, Beijing, China; ^3^ Department of Bone and Joint Surgery, Peking University Shenzhen Hospital, Shenzhen, China

**Keywords:** EZH2, T cells, T cell differentiation, T cell activation, antitumor immunity

## Abstract

Enhancer of zeste homolog 2 (EZH2) is the catalytic subunit of polycomb repressive complex 2 (PRC2), which regulates downstream gene expression by trimethylation of lysine 27 in histone H3 (H3K27me3). EZH2 mutations or overexpressions are associated with many types of cancer. As inhibition of EZH2 activity could upregulate the expression of tumor suppressor genes, EZH2 has recently become an interesting therapeutic target for cancer therapy. Moreover, accumulating evidence has shown that EZH2 may contribute to the regulation of immune cells, especially T cells. EZH2 regulates T cell development, differentiation, and function, suggesting that EZH2 also regulates immune homeostasis in addition to tumor suppressor genes. Moreover, EZH2 can regulate T cell fate by targeting non-T cell factors such as metabolism, cytokines, and myeloid-derived suppressor cells. The role of EZH2 in this process has not been fully addressed. This review discusses up-to-date research on EZH2-mediated regulation of immunological function and the progress of immunological therapeutic strategies based on this regulation.

## Introduction

Gene expression is epigenetically modified by changes in chromatin, which affects the accessibility of certain genomic loci to transcription enzymes and subsequent transcription. Histones can be methylated at lysine residues by histone methyltransferases that influence the chromatin condensation and thus regulate gene expression. Enhancer of zeste homolog 2 (EZH2) is the catalytic subunit of polycomb repressive complex 2 (PRC2), which can trimethylate H3K27 of the target gene, thereby silencing transcription and regulating cell development, oncogenesis, and stem cell plasticity (1–3). Emerging evidence has suggested that EZH2 also plays some unconventional roles in cancers, such as functioning as a non-histone protein transmethylase or a PRC2-independent transcriptional factor (4–6).

The functions of EZH2 in cancer cells have been extensively studied (7–9). Abnormal EZH2 expression contributes to increased aggressiveness and proliferation in various solid tumors (10–15). Furthermore, EZH2 may be involved in preserving the stemness of cancer stem cells (7–9). Even in non-cancerous cells, ectopic EZH2 expression promotes cell proliferation as well as neoplastic transformation (14, 16). However, missense mutations in EZH2 can also exert a cancer-promoting effect in hematological tumors (17, 18). EZH2 can also affect tumor progression by regulating metabolism (19). In general, EZH2 promotes stemness and proliferation by silencing the expression of transcription factors that determine cell fate.

In addition to focusing on the role and application of EZH2 in cancer cells, studies have also paid attention to the role of EZH2 in the development and function of the immune system. Considering its critical function in epigenetic regulation of target gene transcription, accumulating experimental evidence has shown that EZH2 contributes to the development, proliferation, and function of T cells and may act as an immunomodulatory factor. Abnormal EZH2 expression may be implicated in various immune-related diseases, including autoimmune diseases, infection, graft-*versus*-host disease, and cancer (20–22). In addition to its typical PRC2-dependent histone methyltransferase function, limited evidence has shown that EZH2 may also regulate T cell fate *via* unconventional manners. Further studies on the regulatory function of EZH2 in immunity are beneficial for identifying therapeutic strategies. In this review, we discuss the current knowledge of the functions of EZH2 in T cell development, differentiation, and function and its potential value in cancer treatment and immunotherapy.

## EZH2 in T Cell Development

**Figure 1:**
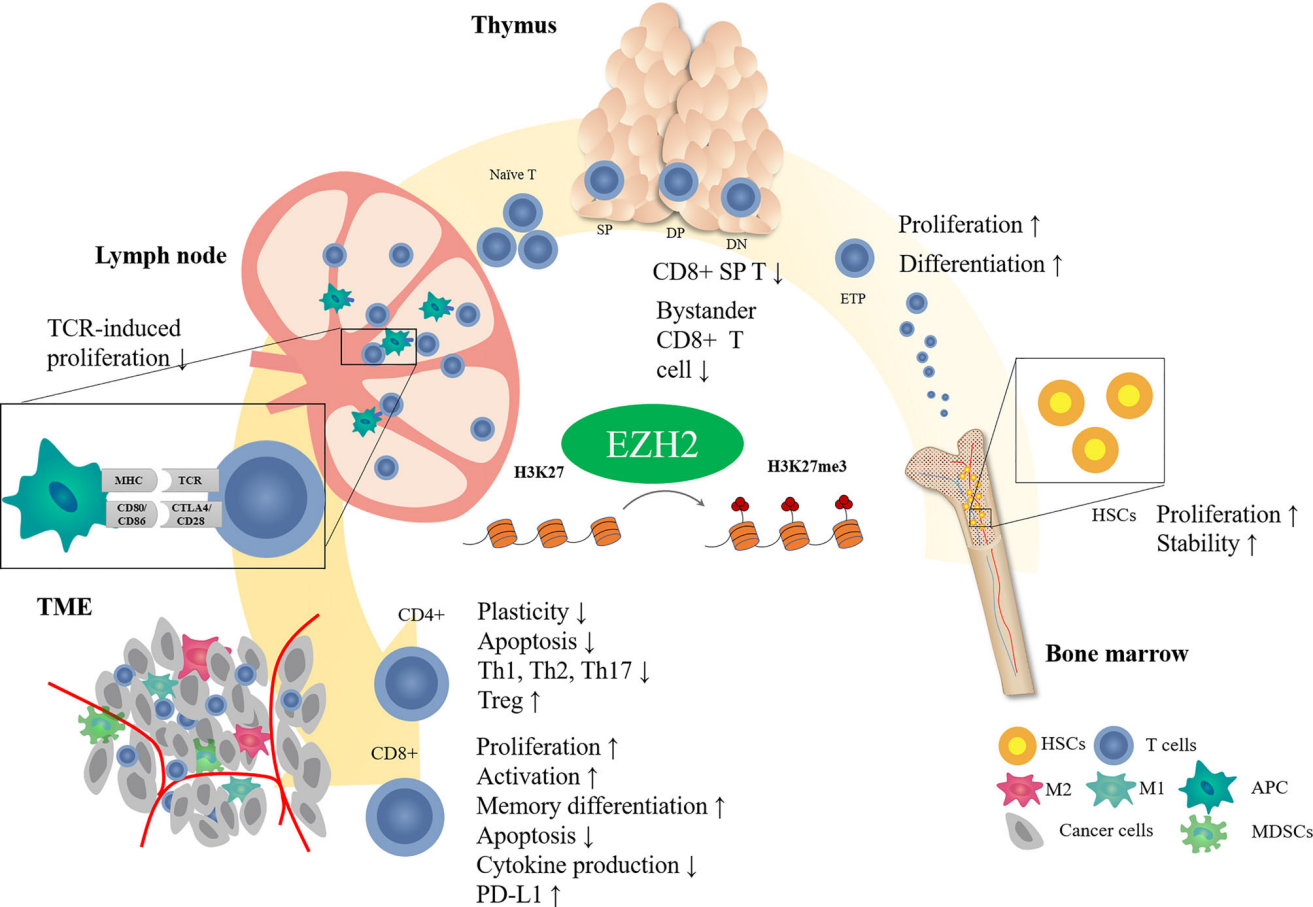
Effect of EZH2 on T cell development. T cells receive antigenic stimulation in lymph nodes after maturation in the thymus and are recruited into the tumor region to exert antitumor effects. EZH2 modifies different stages of T cell development through epigenetic regulatory mechanisms.

EZH2 is important in regulating T cell development and differentiation (**Figure 1**). In hematopoietic stem cells (HSCs), EZH2 plays an essential role in maintaining HSC stemness by negatively regulating cell cycle inhibitors such as Cdkn2 (23, 24). Deletion of EZH2 causes serious myelosuppression and anemia in fetal mice (24). However, hematopoietic phenotypes may not be significantly impacted by EZH2 deficiency because of the compensatory role of EZH1 in adult hematopoiesis (25). Double inactivation of EZH1 and EZH2 impairs the function of bone marrow HSCs (26). Deficiencies in other components of PRC2, such as SUZ12 or EED, can cause similar results (25, 27).

T cells in the thymus stage are also closely regulated by EZH2. EZH2 deficiency is associated with reduced H3K27me3 levels in thymic progenitors and upregulation of expression of the cell cycle inhibitor Cdkn2a. Inactivation of EZH2 in bone marrow cells causes increased arrest of thymocytes in the double negative (DN) phase (28), and the combined loss of EZH2 and Cdkn2a can partially rescue the cell number (28, 29). These results indicate that EZH2-induced methylation regulates thymocyte development by inhibiting cell cycle inhibitors.

Even though EZH2 inactivation impaired thymocyte development, it did not change the production of mature peripheral T cells (30). It suggested that there may be other factors, such as cytokines or transcription factors, restricting the EZH2-mediated regulation of thymocyte development except EZH2. In consistency, T-cell-specific knockout of both EZH1 and EZH2 did not affect the expression of characteristic T cell differentiation or activation markers but reduced T cell numbers slightly in secondary lymphoid organs (30). Interestingly, EZH2-deficient CD8+ thymocyte tended to express surface markers (CD24^lo^, CD44^hi^, and CD122^hi^) of memory-like T cells, also known as bystander T cells (31). However, Vasanthakumar *et al.* reported that neither T-cell-specific inactivation of EZH2, SUZ12, or EED does not alter the αβ or γδ T cell development in thyme (32). Based on the current understanding of EZH2 on T cell development, more studies are needed to uncover the effect of EZH2 on T cell development in thymus.

## EZH2 in the Antitumor Immune Effects of T Cells

### EZH2 in T Cell Activation

TCR recognition is essential for initiating an antitumor immune response. EZH2 plays an unconventional role in TCR stimulation. EZH2 forms a PRC2-like complex and is present in the cytoplasm of T cells. Cytoplasmic EZH2 is localized proximal to TCRβ and contributes to TCR-driven actin polymerization, which promotes interactions between T cells and antigen-presenting cells (30, 33). Immunoprecipitation demonstrated an interaction between EZH2 and CD3_ϵ_, which may form a complex with the TCR-signaling protein Vav1 and the Vav1-associated protein Nck (30). EZH2-deficient T cells maintained their proliferative ability in response to TCR stimulation, while EZH1/2 double-deficient T cells showed reduced IL-2 and IL-2RA expression and failed to proliferate in response to TCR triggering *in vitro*, which could not be rescued by exogenous IL-2 (30). This phenomenon may result from diminished TCR-mediated activation of Erk, which plays a key role in MAPK/Erk pathway-mediated regulation of IL-2 and IL-2RA expression and activation (34). Notably, EZH1/2 double deficiency in T cells showed no impact on Erk phosphorylation or T cell proliferation when stimulated by TCR-independent T cell activation with PMA in combination with ionomycin (30), suggesting the selective role of EZH1/2 in TCR-driven T cell activation. Although they exhibit impaired proliferation, EZH2-deficient CD4+ T cells retain their ability to initiate TCR-induced activation and proliferation *in vitro* (35, 36). Therefore, these results indicate that although EZH2 interacts with CD3, it may not regulate antigen recognition; rather, it may regulate TCR-induced proliferation together with EZH1. Additional studies are needed to confirm the effect of EZH2 on T cell activation in the tumor microenvironment.

### EZH2 in CD8+ T Cells

**Figure 2:**
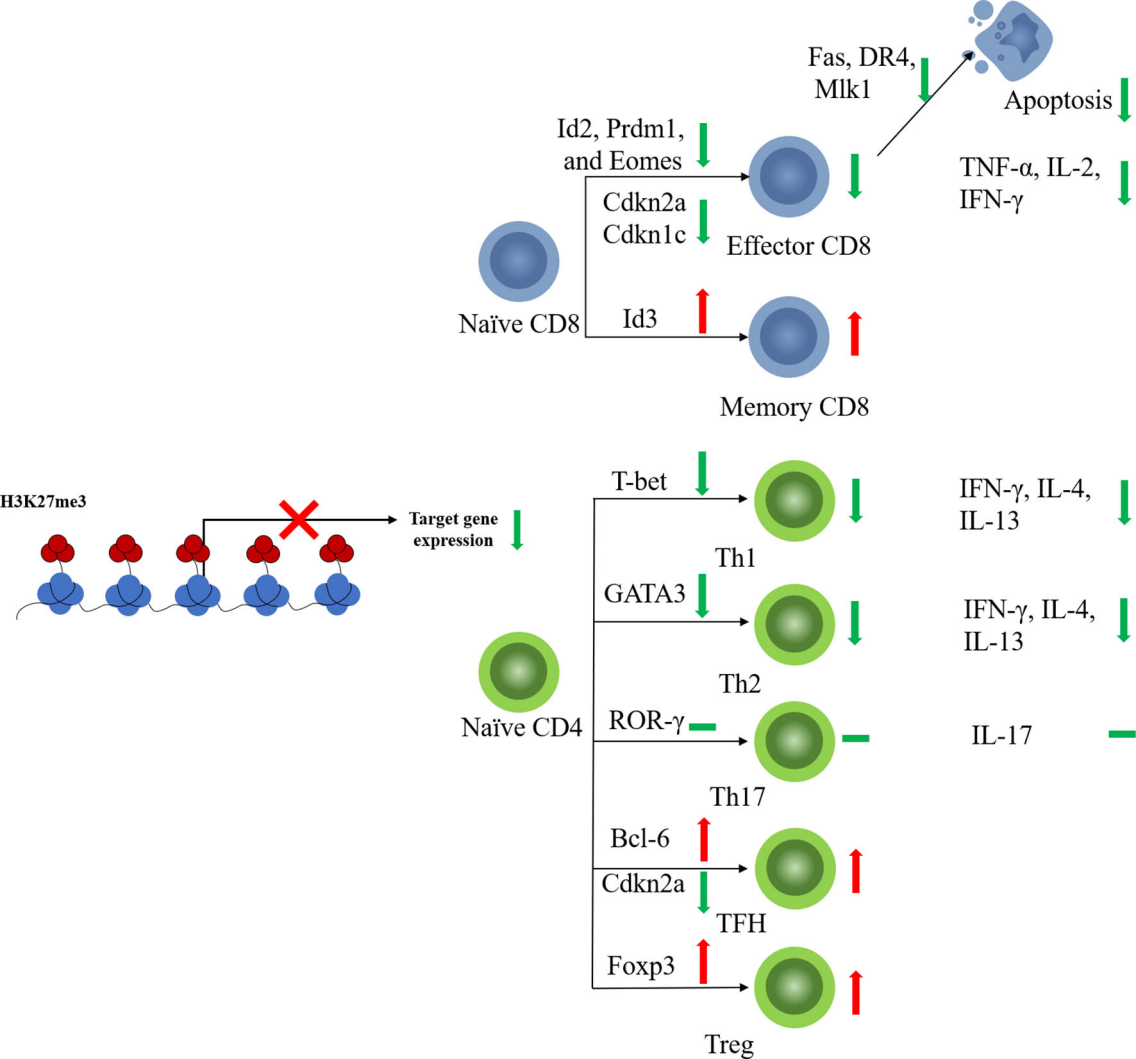
EZH2 function on T cell fates. EZH2 affects differentiation of CD4+ or CD8+ naïve T cells toward effector subtypes by regulating the expression of key transcription factors. EZH2 can also limit the apoptosis of mature effector T cells.

CD8+ T cells play a key role in the defense against tumor cells. Antigen-specific CD8+ T cell responses display a remarkably reproducible pattern of activation, proliferation, differentiation, contraction, and memory cell formation (37). The characteristic epigenetic features of different CD8+ T cell stages have been validated (38). In line with its function in other immune cells, EZH2 regulates the activation, proliferation, and differentiation of CD8+ T cells *via* histone methylation modification (**Figure 2**).

Naïve CD8+ T cells with EZH2 deficiency from thymocyte development (EZH2^fl/fl^CD4Cre^+^) displayed impaired proliferation and increased apoptosis in response to antigen stimulation (39). EZH2 promotes CD8+ T cell proliferation by downregulating the cyclin-dependent kinase inhibitor. Deletion of EZH2 resulted in increased expression of *Cdkn2a* and *Cdkn1c* in activated naïve CD8+ T cells along with reduced levels of H3K27me3 at the loci of these two genes (39). Naïve CD8+T cells are activated and initiate proliferation after T cell receptor (TCR)-antigen stimulation. Consistent with the pro-proliferative role of EZH2, significantly elevated EZH2 protein levels were observed in activated T cells (40).

Besides, EZH2 also regulates naïve CD8+ T cell differentiation. EZH2-deficient CD8+ naïve T cells showed impaired memory cell formation after TCR stimulation (41). Conditional EZH2 deficiency in T cells causes upregulation of expression of pro-effector genes such as Id2, Prdm1, and Eomes upon TCR stimulation (41). Interestingly, Id3, a pro-memory transcription factor, was downregulated with EZH2 deficiency, suggesting that EZH2 activates rather than represses Id3 (41). It has been reported that transcription factors regulating T cell fate (e.g., Tbx21, Prdm1, Eomes, and Irf4) are modified by both repressive H3K27me3 and permissive H3K4me3 histone methylation (42, 43). Decreased H3K4me3 levels were observed at Id3 loci along with decreased H3K27me3 levels (41). EZH2 likely activates Id3 transcription in T cells by regulating the balance between H3K27me3 and H3K4me3, but the underlying mechanism remains unclear (41). These results suggest that EZH2 plays a pro-memory role in the selection process of T cell differentiation under different but shared congenerous epigenetic mechanisms. Moreover, the above function of EZH2 is also regulated by Akt-mediated EZH2 phosphorylation, which results in dysfunctional EZH2 and diminished T memory potential (41), suggesting that the regulatory function of EZH2 in CD8+ T cells is also affected by its posttranscriptional modification state.

CD8+ T cells kill malignant cells by secreting cytokines, such as TNF-α and IFN-γ. Increased evidence indicated that EZH2 may regulate cytokine production by CD8+T cells. EZH2-inactivated naïve CD8+ T cells showed increased production of IFN-γ, interleukin (IL-2), and GZMB after stimulation *in vitro* (39). Therefore, EZH2 appears to inhibit the production of cytokines, especially pro-inflammatory cytokines. However, other studies observed opposite results. Conditional EZH2 knockout in activated CD8+ T cells impaired TNF-α production but produced equivalent percentages of IFN-γ+ and IL-2+ cells compared to those of EZH2^fl/fl^ cells 8 days after viral infection (41). In addition, both CD4+ and CD8+ T cells from the peripheral blood of healthy donors, which have high EZH2 expression levels, also produce multiple effector cytokines, suggesting that EZH2 plays pro-cytokine and polyfunctional roles in CD8+ T cells (44). Considering these contradictory results, the role of EZH2 in cytokine production by CD8+T cells need to be placed in each concrete cancer environment.

When under persistent antigen stimulation, effector CD8+ T cells can develop into terminally differentiated (TE) T cells, which are also known as senescent CD8+ T cells. EZH2 suppresses the expression of pro-memory genes in KLRG1^Hi^IL7R^Lo^ TE effector CD8+ T cells *via* deposition of H3K27me3 at these genes (40). FoxO1 is likely a key regulator that induces the functional change in EZH2. Decreased FoxO1 expression and binding to pro-memory genes were detected in TE CD8+ T cells compared to those in memory T cells. FoxO1 deficiency led to the accumulation of H3K27me3 in the pro-memory genes *Tcf7* and *Bach2* (40), suggesting that FoxO1 may promote and maintain the multipotency of memory T cells by shielding pro-memory genes from H3K27me3 deposition. Deficiencies in either EZH2 or FoxO1 lead to decreased TE cell formation (40). It can be concluded that EZH2 plays variable roles in different stages of CD8+ T cell development: in the early stage after TCR stimulation, EZH2 exerts a memory-promoting effect on CD8+ T cells, whereas in the late stage, EZH2 inhibits pro-memory gene expression and promotes TE cell formation.

The memory T cells are critical for T cell persistence and efficient tumor killing especially for cancer immunotherapy. Differentiated effector CD8+ cytotoxic T cells (CTLs) have a short life span and a tendency for apoptosis (45). EZH2 may play an important role in the regulation of apoptosis-related genes, such as *Fas*, *DR4*, and *Mlk1*, to avoid excessive attenuation of activated T cells (46). EZH2-deficient activated CD8+ T cells exhibit increased expression of apoptosis-related genes as well as elevated rates of apoptosis (39). As for memory CD8+ T cells, although EZH2 may not be required for memory CD8+ T cell formation, increased apoptosis-related gene expression was also observed in sorted EZH2-deficient central memory CD8+ T cells (41), which suggests that EZH2 is essential for memory T-cell persistence.

The studies mentioned above indicated the role of EZH2 in regulating CD8+ T cell function. These results should be considered for the therapeutic design of EZH2 inhibitors, especially in combination with immunotherapy.

### EZH2 Regulates MDSCs Generation and Recruitment

After antigen stimulation, naïve CD4+T cells become non-polarizing Th0 cells and differentiate into distinct subsets of CD4+ T cells, such as Th1, Th2, Th17, regulatory T (Treg) cells, and follicular Th (TFH) cells. The lineage selection of Th0 cells is programmed by transcription factors and cytokines (47). The plasticity of CD4+ T cell differentiation is tightly regulated by the coordination of DNA methylation and histone modifications (48, 49), and the epigenetic mechanisms through which EZH2 regulates CD4+ T cell fate are highly complex (**Figure 2**). First, EZH2 inactivation leads to slight enhancement of CD4+ T cell polarization under short-term culture conditions. With T cell-specific EZH2 knockout, CD4+ T cells showed impaired H3K27me3 levels as well as increased expression of characteristic transcription factors, including T-bet and GATA-3, leading to improved cytokine production and enhanced differentiation of naïve CD4+ T cells into effector Th1 and Th2 cells (35). Consistent with this study, T-cell-specific knockout of EZH2 led to enhanced Th1 and Th2 differentiation but reduced Treg cell differentiation *in vitro* under the respective differentiation conditions for 3 days (50). Enhanced secretion of Th1 and Th2 cytokines, such as IFN-γ, IL-4, and IL-13, was also detected in EZH2-deficient Th1 and Th2 cells (36). Interestingly, EZH2 deficiency enhanced the plasticity of Th0 cells, enabling the transformation of differentiated Th1 cells into Th2 and *vice versa* (36). Second, in long-term culture conditions, this enhancement of effector helper T cells seems to be a kind of “false prosperity”. When stimulation was extended to 6 days, the proportion of EZH2-CKO CD4+ effector (Th0, Th1, Th2, Th17) T cells was decreased compared to that in the wild-type (WT) control (35). Similarly, EZH2-deficient Th1 cells showed an impaired ability to respond to IL-2 after long-term culture for 10 days (50). Consistent with the reduced cell number, upregulated levels of proteins of both intrinsic (tBid and Bax/Bak) and extrinsic (FasL, TNFR1, and TRAIL) apoptotic pathways were present in EZH2-CKO Th0 cells, but not in EZH2^fl/fl^ cells. Inhibition of the apoptotic pathway provided significant but incomplete protection of the survival of EZH2-CKO Th0 cells compared with that of WT cells. These results suggest that EZH2 inactivation of T cells in long-term culture can promote cell death (35). Third, under the condition of chronic infection or tumor, EZH2 deficiency may not be conducive to a normal immune response. Decreases in the percentage and total number of CD4+ T cells were detected in both the spleen and peritoneum in an intraperitoneal parasite infection model, together with a reduced percentage of IFN-γ-producing CD4+ T cells and a poor survival (50). Similarly, mixed-bone marrow chimeric mice harboring WT and EZH2^fl/fl^/CD4-Cre cells showed a significantly reduced proportion of IFN-γ+ Th1 cells compared to that of the control group five days after infection with *Listeria monocytogenes* (35). A further reduction in the number of EZH2-null IFN-γ-producing CD4+ and CD8+ T cells was also observed in chimeric mice (35).

In addition to regulating CD4+ helper T cells, EZH2 can also mediate helper T cells recruitment by affecting chemokine expression. Both genetic and pharmacological inhibition of EZH2 *in vitro* enhanced IFN-γ-mediated induction of Th1-type chemokines, such as CXCL9 and CXCL10, in human ovarian cancer cells (51). Pharmacological EZH2 inhibition *in vivo* also led to increased levels of tumor-producing pro-Th1 chemokines and enhanced recruitment of effector T cells, resulting in reduced tumor volume and prolonged survival time in human ovarian cancers (51, 52). Similarly, in B cell lymphoma cell lines, pharmacological inhibition of EZH2 by EPZ6438 enhanced the expression of CCL17, a chemokine which attracts CCR4+ T cells and facilitates the T-cell response (53).

Few results have been reported regarding the regulatory effects of EZH2 on other helper T cell subsets, such as Th17 or TFH cells. Enhanced Th17 differentiation has also been observed in EZH2-deficient T cells (50). However, only a very slight increase in IL-17 production and RORγ expression was detected in EZH2-CKO CD4+ T cells cultured under Th17 cell-inducing conditions, with reduced EZH2 and H3K27me3 levels at *Il17a* and *Rorc* loci (36). This suggests that the epigenetic mechanisms of EZH2 may be insufficient to regulate Th17 differentiation; other means, such as cytokine production, are also involved in regulating Th17 cell differentiation. TFH cells are another specialized subgroup of CD4+ T cells that prompt B cells to undergo somatic hypermutation, affinity maturation, and transition into plasma cells and memory B cells (54). The promoter of Bcl6, the characteristic transcription factor of TFH cells (55), is associated with H3K27ac rather than H3K27me3, suggesting that EZH2 may not play the key function in this case. However, EZH2 is recruited by *Tcf1* to directly activate Bcl6 transcription, and the function of Bcl6 requires EZH2 phosphorylation at Ser21, suggesting the unconventional role of EZH2 in regulating TFH cell fate (56). Furthermore, EZH2 represses *Cdkn2a* expression through H3K27me3, which regulates TFH cell proliferation and apoptosis (56). Therefore, EZH2 may regulate TFH differentiation, proliferation, and function in both PRC2-dependent and -independent manners.

Taken together, EZH2 inactivation enhanced the recruitment of T cells into the tumor region by upregulating chemokine expression and promoted naïve CD4+ T cells to differentiate into effector Th cells in the short term. However, EZH2 inactivation also impaired the long-term proliferative potential of T cells by activating multiple death pathways, including but not limited to apoptosis, and eventually impaired the antitumor immune response. Therefore, even though EZH2 impairs antitumor immune response by suppressing Th cell differentiation, inhibiting its plasticity and downregulating tumorous chemokine production, it also promotes the stability of effector T cells under tumor conditions.

### EZH2 in Treg Cells

Treg cells are central inhibitory regulators in antitumor activity. Generally, Treg cells refer to a subgroup of CD4+ T cells that express the transcription factor Foxp3, which is a typical functional transcription factor of Treg cells. Foxp3 expression can be inhibited by STAT1 and STAT6, which are downstream activators of IFN-γ and IL-4, respectively (57). EZH2-deficient naïve T cells showed an impaired ability to differentiate into induced Treg (iTreg) cells under Treg-inducing conditions *in vitro*, which could be rescued by combining blockades of IFN-γ and IL-4 (50). Considering that T cell-specific inactivation of EZH2 enhances the production of proinflammatory cytokines, such as IFN-γ, in non-polarizing Th0 cells (35, 50), EZH2 may indirectly affect iTreg differentiation through aberrant production of cytokines, such as IFN-γ and IL-4, rather than through direct epigenetic regulation. However, whether inactivation of EZH2 increases the sensitivity of non-polarizing Th0 cells to cytokines remains unclear and warrants confirmation by further experiments.

In addition to regulating Treg differentiation, EZH2 is required for Treg activation, as evidenced by a strongly elevated expression of EZH2 in CD44^hi^CD62L^lo^-activated Treg cells compared to that in naïve or resting Treg cells (58). In tumor conditions, tumor-infiltrating lymphocytes have a higher proportion of total and activated Tregs than normal tissues (59). EZH2 expression is also upregulated in tumor-infiltrating Treg cells compared to that in effector T cells or Treg cells in lymph nodes (60). Increased H3K27me3 levels have been detected in activated Treg cells (61). Consistent with this finding, co-immunoprecipitation experiments showed that EZH2 forms complexes with Foxp3 in CD62L^lo^-activated Treg cells but not in resting Treg cells (58), suggesting that EZH2 plays a role in CD44^hi^CD62L^lo^-activated Treg cells by binding to Foxp3. Furthermore, similar differentially expressed genes were detected between EZH2- and Foxp3-deficient CD62L^lo^-activated Treg cells, but not in resting Treg cells (61), suggesting that the regulatory effect of activated Treg cells is mediated by the cooperation of EZH2 and Foxp3. These results also suggest that EZH2 may cooperate with Foxp3 under the non-classical regulatory mechanism.

Considering the important role of Treg cells in maintaining immune homeostasis, the loss of EZH2 may cause dysregulation of immune homeostasis (61). Foxp3-specific EZH2 deficiency increased T cell activation and infiltration in lymphoid organs, but it reduced the frequency in non-lymphoid tissues where effector Treg cells are resident (62). Conditional knockout of EZH2 in Treg cells causes impaired immune tolerance, including various symptoms of autoimmunity (such as reduced weight, hair loss, and scaly tails), extensive infiltration of mononuclear immune cells in non-lymphoid tissues (such as the lungs, liver, pancreas, and kidneys), and a reduced lifespan in mutant mice (61). Furthermore, Foxp3-specific EZH2 inactivation led to enhanced production of pro-inflammatory cytokines as well as reduced tumor size in murine bladder cancer (63). These results further confirmed that EZH2 is responsible for Treg cell activation, which is critical for the immunosuppressive function that maintains immune homeostasis.

## EZH2 Compromises Antitumor Immune Effects of T Cells

In addition to its direct regulatory effect on T cells, EZH2 may also induce immune escape to some extent by affecting other factors in the TME, such as immune checkpoints, MDSCs, and cytokines.

### EZH2 Regulates Immune Checkpoint Expression

Tumor cells in the TME may negatively influence the immune system *via* EZH2, thus impairing immune cell function and achieving immune escape. For example, tumor cells can express immune checkpoint ligands and bind to their receptors on T cells, thus suppressing antitumor immunity. Therefore, immune checkpoints may be potential therapeutic targets for the epigenetic regulation of EZH2. PD-1 and its ligand, PD-L1 (CD274), are the most well-known immune checkpoint targets (64). The PD-L1 expression in tumor-induced exhausted CD4+ T cells was found to be controlled by the interplay of chromatin remodeling SWI/SNF and PRC2 complexes, suggesting that EZH2 is involved in regulating PD-L1 expression in exhausted CD4+ T cells (65). However, contradictory results have been found regarding the regulatory effect of tumor EZH2 on PD-L1 expression (66, 67). A negative correlation between EZH2 and PD-L1 expression was observed in hepatocellular carcinoma and prostate cancer, which may result from the upregulation of H3K27me3 levels in the promoters of *CD274* and *IRF1* (66, 68). Therefore, inhibition of EZH2 function may enhance PD-L1 expression in tumor cells. Consistent with this hypothesis, both genetic and chemical inhibition of EZH2 in prostate cancer organoids repressed the H3K27me3 levels and upregulated the endogenous dsRNA levels. Accumulated endogenous dsRNA can activate the interferon-response pathway and upregulate tumor expression of PD-L1 in a STING-dependent manner (68). However, in another lung adenocarcinoma model, EZH2 expression was positively correlated with PD-L1 expression (67), but the mechanisms are uncertain. Although the exact regulatory effect of EZH2 on immune checkpoints remains unclear, EZH2 inhibitors in combination with immunotherapy seem to be a viable antitumor treatment strategy. Treatment with epigenetic modulators increases effector T cell infiltration, represses tumor progression, and promotes the therapeutic efficacy of PD-L1 checkpoint blockade in prostate or head and neck cancer (68, 69). Pharmacological inhibition of EZH2 also inhibits tumor growth and improves the efficacy of anti-CTLA-4 treatment in bladder cancer (63).

### EZH2 Regulates MDSC Generation and Recruitment

MDSC is another cell population that exhibits an inhibitory regulator role in antitumor activity. Evidence has shown that MDSCs can be induced by EZH2-mediated epigenetic regulation. Upon induction of monocytic (M)-MDSCs *in vitro*, a gradual loss of EZH2 and H3K27me3 was observed along with a gradual expansion of the CD11b^+^Gr-1^+^ M-MDSC population (70, 71), suggesting that loss of EZH2 activity may be responsible for MDSC generation. Pharmacological inhibition of EZH2 enhanced MDSC generation in both tumor and non-tumor models. GSK343 and GSK126 both inhibit the histone methyltransferase activity of EZH2 without affecting its protein expression. In an inflammatory bowel disease model, GSK343 treatment caused the elevation of MDSCs in the colonic lamina propria and peripheral blood but did not alter the expression of MDSC-related chemokines, suggesting that EZH2 inhibition by GSK343 enhanced M-MDSC generation without affecting its chemotaxis (70). Similar results were observed in an MC38 mouse colon cancer tumor model. EZH2 inhibition by GSK126 was reported to promote M-MDSC formation, decrease T cell proliferation, and increase T cell apoptosis, leading to impaired antitumor immunity (71). Consistent with the results observed in the inflammatory bowel disease model, GSK126 treatment increased the frequencies and numbers of granulocyte/macrophage progenitors, which are also progenitors of MDSCs, within the hematopoietic progenitor cell (HPC) population, suggesting that GSK126-treated HPCs had an enhanced potential to differentiate into myeloid cells (71). Additionally, proliferation and apoptosis of MDSCs were unaffected by treatment with GSK126, suggesting that differentiated MDSCs are rarely regulated by EZH2, which is consistent with their low EZH2 and H3K27me3 levels (70, 71). Mechanistically, activation of the Jak-STAT and tumor necrosis factor (TNF) signaling pathways, which are known to regulate MDSC production (72, 73), was observed in GSK343-induced M-MDSCs *in vitro*, suggesting that GSK343 may enhance M-MDSC generation by activating Jak-STAT and TNF signaling (70). Additionally, EZH2 was reported to activate NF-κB as a PRC2-independent coactivator of transcriptional factors in ER-negative basal breast cancer (74). In hepatocellular carcinoma, cell cycle-related kinase (CCRK) enhanced IL-6 production *via* the abovementioned EHZ2/NF-κB signaling and induced increased accumulation of polymorphonuclear (PMN)-MDSCs (75), which suggests another mechanism by which EZH2 regulates MDSC generation. Although EZH2 inhibitors can enhance MDSC production *via* various pathways, not all EZH2 inhibition can induce the enhanced generation of MDSCs. Another EZH2 inhibitor, EPZ0011989, did not change the frequency of either M-MDSCs or PMN-MDSCs in mouse prostate tumor tissue (68). Therefore, further evidence is needed to exclude the influence of tissue origin specificity or different inhibitors.

### Other Immune Subgroups

The regulatory effect of EZH2 on other immune cell subsets can also affect T cell function and antitumor immunity. Inhibition of EZH2 upregulated the expression of NKG2D, an activating receptor required for natural killer (NK) cells (76). Alternatively, EZH2 inhibition was also reported to induce the expression of NKG2D ligands in hepatocellular carcinoma cells (76). Taken together, EZH2 inhibition may promote NK-induced cytotoxicity by affecting both the tumor and NK cells. Tumor-associated macrophages are also an important part of the TME. EZH2 inhibition in glioblastoma induced an increase in M1 markers (iNOS and TNF-α) and reduction in M2 markers (tumor growth factor-β1, -β2, and stabilin-1) in both murine microglia and human peripheral blood mononuclear cell-derived macrophages (77), which is indicative of the pro-M2 function of EZH2. Furthermore, expanded M2 macrophages express increased levels of anti-inflammatory cytokines and play an immunosuppressive role in the TME (78).

## Environmental Factors in the Regulation of T Cells by EZH2

**Figure 3:**
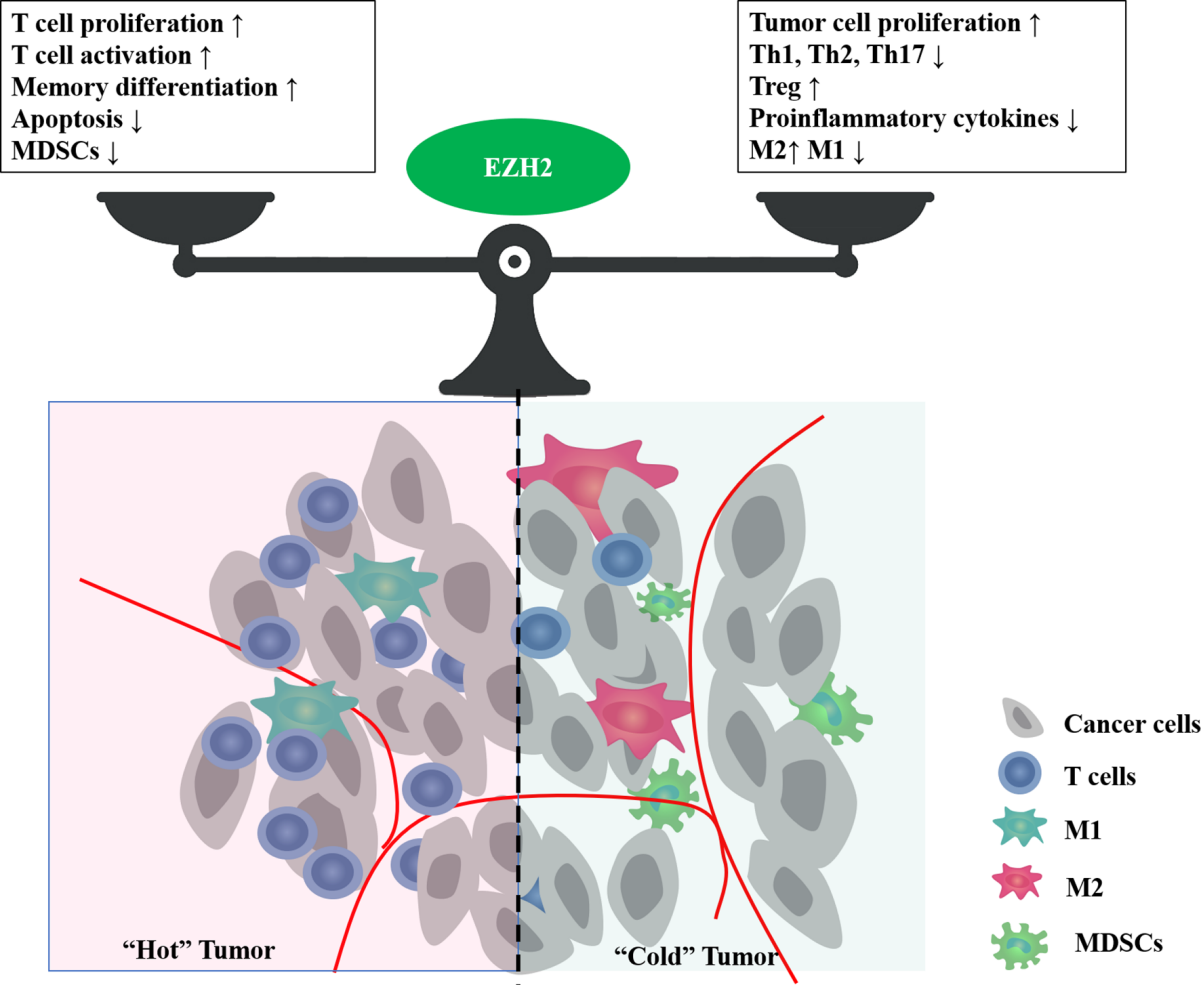
The bidirectional effect of EZH2 on tumor immune microenvironment. EZH2 can regulate multiple cellular components in the tumor microenvironment, such as cancer cells, T cells, macrophages, and myeloid-derived suppressor cells (MDSCs). Major phenotypes associated with EZH2 function in balancing the tumor immune microenvironment.

Tumor development relies on the TME, such as hypoxia, nutritional restrictions, and metabolic reprogramming. These environmental factors are shaped jointly by multiple types of cells, including tumor cells, tumor vessels, immune cells, and stromal cells, and change dynamically with tumor progression. EZH2 expression is influenced by the TME, and conversely, it is involved in shaping the TME (**Figure 3**).

### Hypoxia and Hypoxia-Inducible Factor (HIF)-1α

HIF-1α is a key downstream immunoregulator of EZH2. In cancer cells, under normoxia, EZH2 is recruited directly to the HIF-1α promoter and inhibits HIF-1α expression, whereas under hypoxia, this inhibitory effect is weakened, leading to unregulated HIF-1α expression (79). EZH2 knockdown further upregulates HIF-1α expression under hypoxic conditions (79). By contrast, studies have shown that HIF-1α can upregulate EZH2 expression in tumor models (80, 81). Activation of HIF-1α can recruit MDSCs to the primary tumor site by enhancing the expression of chemokine (C–C motif) ligand 26 in tumor cells (82), which can further inhibit antitumor T cell effector function. This may be one reason why EZH2 inhibition by GSK126 led to MDSC recruitment in the TME (71). The regulatory function of EZH2 on HIF-1α in immune cells has not been proven, but this effect, if present, may have a profound effect on the immune system. HIF-1α is a key regulator of T cell survival, proliferation, and effector function (83–85), which may be due to the hypoxic environment in the bone marrow and thymus. Therefore, upregulation of HIF-1α expression by EZH2 inhibition can protect T cells from hypoxia-induced apoptosis during early developmental stages. However, CD73, a HIF-1α-regulatory molecule that is mainly expressed on Treg cells or some tumor cells (86), regulates extracellular adenosine production. Adenosine plays an immunosuppressive role in the TME mainly through the adenosine 2A receptor on T cells (87–89). Thus, EZH2 inhibition in a hypoxic TME can further enhance extracellular adenosine production *via* the hypoxia/HIF-1α/adenosinergic pathway and impair antitumor immunity. Therefore, the bidirectional effect of HIF-1α at different stages of T cell development may be one reason for the complexity of the regulatory effect of EZH2 on T cells.

### Metabolism

T cell fate is closely related to metabolism, which is highly regulated by epigenetics. Some EZH2-regulatory metabolic targets have been reported to have immunoregulatory effects. For instance, in lung cancer, EZH2 can suppress Lat1 and promote methionine uptake, which ultimately upregulate the synthesis of s-adenosine methionine (SAM), an important transporter of one carbon unit (90). SAM can induce T cell exhaustion *in vitro*, and interference with the synthesis of SAM *via* knockdown of MAT2A, a key SAM-producing enzyme, results in the inhibition of T-cell exhaustion in a mouse hepatocellular carcinoma model (91). Therefore, EZH2 inhibition can repress SAM synthesis and counteract its exhaustion-promoting effect. Additionally, SIRT6, a histone/protein deacetylase, regulates critical signaling pathways related to multiple metabolic processes, including PI3K/Akt/mTOR (92), HIF-1α (93), and NF-κB (94), which are also closely related to the immune response. SIRT6 is reported to be silenced by EZH2 (95). Thus, we can consider a SIRT6-participating pathway in the EZH2-regulatory function of the immune response. Furthermore, EZH2 functions in restricting glycolysis in T cells within the TME through the glycolytic pathway, which leads to reduced proinflammatory cytokine expression and poor survival of CD8+ T cells, thus ultimately increasing the tumor burden and metastatic potential of the melanoma model (44). Moreover, T cell functions in the TME are closely regulated by lipid metabolism (96). In an MC38 murine colon cancer model, high-fat diet (HFD)-induced obesity impaired CD8+ T cell function in the TME and led to poor outcomes, which resulted from distinct metabolic adaptations to obesity between the tumor and CD8+ T cells (97). Tumor cells increase fat uptake of the HFD and lead to altered fatty acid partitioning in HFD tumors, impairing CD8+ T cell infiltration and function (97). EZH2 is an important regulator of lipid metabolism. In glioma harboring telomerase reverse transcriptase (TERT) promoter mutations, TERT and EZH2 cooperate with fatty acid synthase (FASN) and induce fatty acid accumulation. Additionally, siRNA-mediated EZH2 knockdown reduced the abundance of intracellular fatty acids (98). EZH2 promotes fatty acid synthesis and lipid accumulation through the TERT-EZH2 network. However, under different experimental conditions, such as in hepatocyte cell lines (99) or breast cancer (100), inhibition of EZH2 induces lipid accumulation. This discrepancy may arise because of differences in tissue specificity. Although the effect of EZH2 on lipid metabolism in different cells or models varies, treatments targeting EZH2 have the potential to reshape lipid composition in the TME, which could further affect T cell function in the TME. More direct evidence is needed to verify the regulatory effect of EZH2 on T cells through metabolism.

## Discussion

As the core catalytic subunit of PRC2, EZH2 plays a crucial role in the epigenetic control of chromatin accessibility for gene transcription *via* histone trimethylation. In addition to its known onco-promoting effects, EZH2 in the TME can influence the immune response and prognosis through its interaction with immune cells. This review focuses on T lymphocytes, which play an essential role in immune homeostasis. EZH2 regulates multiple aspects affecting the immune ability of lymphocytes, including maturation, differentiation, proliferation, function, and apoptosis. Abnormal EZH2 expression is closely associated with aberrant T-cell differentiation and function.

In clinical data, the roles of EZH2 in regulating T cell-mediated antitumor immunity can be complicated. EZH2 inactivation may activate T cell function in the short term, but it leads to increased apoptosis and impaired function in the long term, resulting in an impaired antitumor immune response. However, specific EZH2 deficiency in immunosuppressive cells, such as Treg cells, can lead to an enhanced immune response. A case study also confirmed the bidirectional complex effects of EZH2 inhibitors on the immune system (101). In a patient with metastatic, poorly differentiated chordoma, clinical use of EZH2 inhibitor EPZ6438 resulted in a significant increase in intratumoral and stromal infiltration by proliferative (high Ki-67) CD8+ T cells, Foxp3+ regulatory T cells, and immune cells expressing checkpoint regulators PD-1 and LAG-3 (101), which meant both immuno-activating and suppressive effects. In patients with ovarian cancer, high rate of EZH2+ CD8+ T cells positively affect cancer survival (44). However, systemic overexpression of EZH2 was significantly associated with impaired effector memory CD8+ T cell infiltration and poor survival in patients with hepatocellular carcinoma (102). These results suggest that EZH2 exerts opposite effects in tumor cells and T cells on antitumor immunity. Therefore, it is an urgent problem to explore how to amplify the immuno-activation effect of EZH2 inhibitors while avoiding their immuno-suppressive effect, to bring more clinical benefits. Some clinical trials are exploring the feasibility of EZH2 inhibitors in combination with immunotherapy for lymphoma and some solid tumors (NCT02220842, NCT03854474, NCT04407741, NCT03525795). However, most of them are phase I/II studies with insufficient data feedback. It is still not enough to analyze the effect of EZH2 inhibitors on immune cells, or to determine whether EZH2 inhibitors can be combined with immunotherapy to provide adequate clinical benefit.

Additionally, the wide range of EZH2 targets also contributes to the complexity of its effect in the TME. Generally, chemical inhibition of EZH2 by an inhibitor will influence both tumor and immune cells in different aspects, including proliferation, apoptosis, metabolism, and cytokine as well as chemokine production. These nonspecific effects may be important barriers to the clinical application of EZH2 inhibitors (103). More research is needed to further reveal the effect, mechanism, and selectivity of different subgroups of EZH2 inhibitors on T cells. The following issues should be resolved (1): how EZH2 can be targeted in specific subgroups or differentiation stages of T lymphocytes (2); how the functional pattern of EZH2 on both tumor cells and T cells changes with tumor progression (3); how combination therapy strategies can be used to eliminate or weaken the adverse effects of EZH2 targeting; and (4) whether targeting EZH2 in combination with immunotherapy could be a new strategy for antitumor therapy. In terms of high tumor heterogeneity, existing studies have only revealed the tip of the iceberg. Further understanding of the mechanisms underlying the epigenetic effects of EZH2 on the development, differentiation, and immune function of T cells might provide new strategies for the combined or synergistic treatment of cancer as well as other immune-related diseases.

## Author Contributions

JH and JZ contributed equally to this work. JY and LX conceived of the article. JH, JZ, ZG, LC,TZ and JY collected related studies and drafted the manuscript. LX, JY, JW and JH were responsible for the final review of the manuscript. All authors contributed to the article and approved the submitted version.

## Funding

This study was supported by the National Natural Science Foundation of China (No. 81972966 and No. 82072870) and the Youth Program of the Beijing Municipal Natural Science Foundation (7204328).

## Conflict of Interest

The authors declare that the research was conducted in the absence of any commercial or financial relationships that could be construed as a potential conflict of interest.

## Publisher’s Note

All claims expressed in this article are solely those of the authors and do not necessarily represent those of their affiliated organizations, or those of the publisher, the editors and the reviewers. Any product that may be evaluated in this article, or claim that may be made by its manufacturer, is not guaranteed or endorsed by the publisher.

## Abbreviations

EZH2, enhancer of zeste homolog 2; PRC2, polycomb repressive complex 2; HSCs, hematopoietic stem cells; WT, wild-type; CTLs, cytotoxic T cells; TME, tumor microenvironment; MDSCs, myeloid-derived suppressor cells; NK, natural killer; TCR, T cell receptor; HIF, hypoxia-inducible factor; Th, helper T; TFH, follicular helper T; Treg, regulatoty T; HPC, hematopoietic progenitor cell; TNF, tumor necrosis factor; CCRK, cell cycle-related kinase; PMN, polymorphonuclear; SAM, s-adenosine methionine.
